# Identifying barriers to outpatient appointment attendance in patient groups at risk of inequity: a mixed methods study in a London NHS trust

**DOI:** 10.1186/s12913-024-10947-8

**Published:** 2024-04-30

**Authors:** Beatrice Sung, Fiona O’Driscoll, Alice Gregory, Kate Grailey, Hannah Franklin, Sharon Poon, Anna Lawrence-Jones, Leila Shepherd, Clare McCrudden, Bob Klaber, Chris Pavlakis, Ara Darzi, Sarah Huf

**Affiliations:** 1 University of Bath, Bath, UK; 2 Helix Centre, Institute of Global Health Innovation, Imperial College London, Room 1035/7, QEQM Wing, St Mary’s Campus, London, W2 1NY UK; 3 Imperial College Healthcare NHS Trust, London, UK; 4 Institute of Global Health Innovation, Imperial College London, London, UK; 5 National Institute for Health and Care Research (NIHR) North West London Patient Safety Research Collaboration (PSRC), London, UK; 6 Imperial College London, London, UK

**Keywords:** Health inequity, Health inequality, Outpatient, Appointment, Behavioural science, Design, Public involvement, Ethnic minorities, Deprivation, Underserved groups

## Abstract

**Background:**

There is significant health inequity in the United Kingdom (U.K.), with different populations facing challenges accessing health services, which can impact health outcomes. At one London National Health Service (NHS) Trust, data showed that patients from deprived areas and minority ethnic groups had a higher likelihood of missing their first outpatient appointment. This study’s objectives were to understand barriers to specific patient populations attending first outpatient appointments, explore systemic factors and assess appointment awareness.

**Methods:**

Five high-volume specialties identified as having inequitable access based on ethnicity and deprivation were selected as the study setting. Mixed methods were employed to understand barriers to outpatient attendance, including qualitative semi-structured interviews with patients and staff, observations of staff workflows and interrogation of quantitative data on appointment communication. To identify barriers, semi-structured interviews were conducted with patients who missed their appointment and were from a minority ethnic group or deprived area. Staff interviews and observations were carried out to further understand attendance barriers. Patient interview data were analysed using inductive thematic analysis to create a thematic framework and triangulated with staff data. Subthemes were mapped onto a behavioural science framework highlighting behaviours that could be targeted. Quantitative data from patient interviews were analysed to assess appointment awareness and communication.

**Results:**

Twenty-six patients and 11 staff were interviewed, with four staff observed. Seven themes were identified as barriers – communication factors, communication methods, healthcare system, system errors, transport, appointment, and personal factors. Knowledge about appointments was an important identified behaviour, supported by eight out of 26 patients answering that they were unaware of their missed appointment. Environmental context and resources were other strongly represented behavioural factors, highlighting systemic barriers that prevent attendance.

**Conclusion:**

This study showed the barriers preventing patients from minority ethnic groups or living in deprived areas from attending their outpatient appointment. These barriers included communication factors, communication methods, healthcare the system, system errors, transport, appointment, and personal factors. Healthcare services should acknowledge this and work with public members from these communities to co-design solutions supporting attendance. Our work provides a basis for future intervention design, informed by behavioural science and community involvement.

**Supplementary Information:**

The online version contains supplementary material available at 10.1186/s12913-024-10947-8.

## Background

Health inequity, defined as avoidable and unfair differences in health access, outcomes and experience between groups or populations, is a major issue faced by patients globally [1, 2] and in the United Kingdom (U.K.) [3]. This inequity is long-standing and widespread, for example with racial inequity resulting in Black women having a four-fold increase in maternal mortality rates and Asian women having a two-fold increase, compared to white women [4]. Inequity has also been demonstrated in data arising during the COVID-19 pandemic, with the risk of dying once diagnosed with COVID-19 higher among people living in the most deprived areas compared to the least [5]. In current national policy from National Health Service (NHS) England [6] and government [7], there is a clear focus on reducing health inequalities, a term often used interchangeably with health inequity [6–8]. Health equality is often presented as synonymous with health equity, where people with equal health needs are treated equally [9]. However, health equity identifies the requirement to treat people with unequal needs differently, for them to have an equal health outcome [9]. NHS England’s Core20PLUS5 framework outlines several population groups that should be considered when designing actions to reduce healthcare inequalities, such as the most deprived 20% of the population and ethnic minority communities [6].

First outpatient appointments are a vital way of accessing secondary care and managing complex health problems, acting as a gateway into diagnostics, care pathways and treatments [10]. Recording whether a patient “Did Not Attend” (DNA) their appointment is a key measure for health services to track patient pathways and service utilisation [11]. Appointments that are reported as DNAs reflect those that are missed without the patient cancelling or rescheduling beforehand. A focus on reducing the rate of those who did not attend new outpatient appointments has the potential to improve health outcomes by providing patients with earlier diagnosis and access to specialist expertise and treatments [11–14]. A patient’s history of missing an outpatient appointment is a factor that may contribute to future non-attendance [15], showing the significance of this healthcare interaction. Overall, this will ensure long-term management of chronic diseases and improve health outcomes.

One aspect of health equity is the ability of different groups or populations to be supported in accessing a range of healthcare services, such as outpatient appointments. It has been shown that some patient groups are more likely to not attend an outpatient appointment, with national data sources showing that patients from more deprived areas [16] and minority ethnic groups [17, 18] were more likely to not attend their appointments. This inequity means certain populations may struggle to access healthcare due to various barriers, despite initiatives to improve service utilisation. These efforts include NHS Trusts proactively contacting patients from minority ethnic groups and deprived areas with offers of support for travel costs or parking before their appointment [17] and offering parents of children living in the most deprived areas free transport to appointments [19]. Existing literature has highlighted the barriers in attending outpatient appointments that can lead to non-attendance. A systematic review of studies reporting reasons for non-attendance found that “forgetfulness” was a common cause, as were health-related barriers, timing and compatibility issues, administrative errors and miscommunication, financial issues, and perceived negative outcomes of attending [20]. However, the impact of each barrier is reported variably – for example, forgetting to attend an appointment contributes to between 8 and 45% of DNAs [20]. This suggests that non-attendance is complex, and the barriers that different individuals face can depend on a multitude of factors. Communities facing inequity may experience different barriers or the same barriers but to varying extents. The literature suggests that patients from more socioeconomically deprived backgrounds experience more transport-related issues [20] compared to those from less deprived backgrounds, which may be due to the cost or reliability of public transport. To help individuals at risk of inequity access to healthcare services, the specific barriers they face should be investigated. Corresponding solutions should be co-designed with people from these communities to ensure interventions are tailored to their experiences and circumstances. This is required given the evidence that a “one size fits all” approach to supporting patients attend their appointments is not suitable [21].

Data from one NHS Trust in the U.K. highlighted the presence of inequity in their outpatient attendance rates. In preliminary work, this Trust found that there was a statistically significant difference in proportion of DNA rates for outpatient appointments based upon both deprivation and ethnicity in 11 clinical specialties. Patients whose ethnicity was recorded as “Black”, “mixed” or “other”^1^ or who lived in an area scoring quintile 1 on the Index of Multiple Deprivation (IMD) (most deprived), were up to 50% more likely to not attend their outpatient appointment compared to patients with “White – British” ethnicity or who lived in the least deprived areas (IMD quintile 5).

These data created the rationale for this study – to explore the barriers to attendance in these patient groups identified as being more likely to DNA an outpatient appointment. Given the existing evidence showing that many barriers to attendance are related to wider healthcare and systemic issues, this was also evaluated [11, 20].

Specific aims were to:

1. Understand the barriers to attendance at outpatient appointments faced by patients at risk of inequity based on ethnicity or the deprivation of where they live.
2. Explore the systemic factors that might contribute to DNAs.
3. Assess patient appointment awareness and the communication methods received by patients.

## Methods

### Study design

This project was undertaken as a mixed methods service evaluation in line with the outcomes for the Health Research Authority (HRA) decision tool [23] and received the relevant approvals from the clinical governance team at the study site (reference number 830). Qualitative methods, including semi-structured interviews and observation were used to identify barriers to attendance and explore systemic factors. The interview discussion guides included primarily semi-structured questions that were flexible and facillitated the gathering of rich insights and experiences. Structured, closed questions method were additionally incorporated to quantitatively evaluate appointment awareness and communication methods for patients who had missed their appointment. The study is reported in line with the Standards for Reporting Qualitative Research (SRQR) [24].

### Study setting

Five clinical specialties within outpatient services at the study site were selected as the study setting, along with the Patient Service Centre (PSC). The five clinical specialties were ophthalmology, gastroenterology, colorectal surgery, plastic surgery, and ophthalmology; chosen due to the fact they were high-volume specialties and had a statistically significant disparity in outpatient attendance when considering ethnicity and deprivation. The PSC provides all centralised outpatient appointment booking services and receives inbound patient appointment queries and was therefore included given its importance in the outpatient appointment journey.

### Participants

Patient participants included those who had not attended a recent first appointment at one of the five specialties, and whose ethnicity was recorded in the electronic healthcare record (EHR) as ‘Black’, ‘mixed’ or ‘other’ [25] or whose address had the highest level of deprivation (IMD quintile 1). Participants were identified using appointment data provided by the Trust Business Intelligence (BI) team. Staff participants worked in the outpatient department of one of the five clinical specialties or the PSC. They were identified based on their role and its relevance to the project aims. All staff interviewed had administrative, operational or coordinator roles in an outpatient department, and were involved in coordinating and booking an outpatient appointment for a patient. Staff and patients were recruited using a purposive sampling technique, to ensure the participant groups were reflective of the target populations.

### Data collection

Data collection was carried out from March to April 2023. Two separate semi-structured discussion guides (provided as additional files) were developed based on the literature review and using the theoretical domains framework (TDF) as a behavioural science framework [26]. A behavioural science framework was chosen to underpin the interview discussions, given the study’s focus on understanding the determinants of patient behaviours with respect to them attending their outpatient appointment. It was anticipated that this understanding could be utilised in future work to design bespoke interventions that support attendance at outpatient appointments for groups facing inequity. The TDF is a behavioural science framework that combines different theories of behavioural change and provide researchers with 14 ‘domains’ which summarise different factors influencing behaviour. These domains cover different influences of behaviour, such as knowledge, skills and social identity [26]. The TDF was used to develop the discussion guide to focus the questions on potential behaviours affecting attendance. The discussion guides were reviewed by the research team and developed further as part of the team’s public engagement and involvement work. Feedback from a member of the project’s Public Steering Group (PSG) was incorporated. The PSG member (MH) suggested changes such as explaining the purpose of the phone calls at the beginning of the call with the patient before their details are confirmed, to address power dynamics for those with a history of trauma or negative lived experiences with authorities. Interview findings were used to iterate the discussion guides during the data collection period.

All patient interviews were conducted in English by team members using a hospital phone to contact patients on the phone number(s) recorded on the EHR. Attempted contact was made up to 3 times and all calls were made during working hours of 9am – 5pm, Monday – Friday. Each patient interview was carried out by an interviewer (AG) in a private, quiet room at the NHS Trust, with another member of the research team present acting as note-taker (FO) and transcribing notes verbatim. Verbal informed consent was gained prior to carrying out the interview after explaining the purpose of the study. The interviews were planned to last approximately 10 to 15 min given their unscheduled nature for participants.

To further explore the systemic factors that might contribute to DNAs, staff interviews were carried out either on video call or in-person at the staff member’s hospital location of work and lasted 30 min to one hour. Verbal informed consent was gained prior to carrying out the interview or observation. Where staff were also observed, this was carried out in-person during the interviews and varied depending on the staff role. For staff working in the clinical specialties, observations were carried out by asking them to demonstrate administrative processes and tasks described during the interview that are involved in coordinating and booking a patient’s appointment. Staff working in the PSC were observed by listening to staff respond to incoming patient calls and observing them carrying out their work in booking or rescheduling a patient’s appointment. For most staff interviews, there was one interviewer (AG) and one note-taker (FO) transcribing notes, except for the staff observations at the PSC where both researchers (AG and FO) interviewed and observed separate staff members due to the individual nature of their work receiving inbound patient calls. Recruitment for both patient and staff interviews ended when thematic saturation was reached as determined by the researchers, being the point at which no new themes were being identified through ongoing data capture [27].

To provide a quantitative analysis of whether patients were aware of their missed appointment, at the start of each interview they were asked whether they were aware of the appointment they had missed. If the participant was aware of the appointment, they were then asked what communication method they had received to notify them.

### Data analysis

To analyse patient interview data and identify barriers to attendance, reflexive thematic analysis following an inductive approach was chosen. As outlined below, our thematic analysis process followed a six-phase approach: familiarisation with the dataset, generation of codes, construction of themes by collating codes, and then review, finalisation and naming of themes [28]. All patient interview notes were reviewed by one researcher to become familiar with the dataset (FO). The initial codes were generated by the researcher and themes were constructed by comparing codes and the corresponding data with each other. 10% of patient interview notes were analysed by a second team member (CM) and compared with the initial analysis. An initial framework with themes and subthemes was developed to represent the barriers and issues experienced by patients at risk of inequity based on their ethnicity or the deprivation of where they live.

Following the development of initial themes and subthemes, all staff interview notes were reviewed and coded by one researcher (FO). These data were analysed in a deductive approach with the context of the initial framework to explore the wider systemic issues that may contribute to DNAs. The additional insights gained from interviewing staff about barriers and wider issues added to and expanded the framework. This data triangulation contributed to the development of a comprehensive framework that summarises the issues affecting patients experiencing inequity alongside the wider systemic issues that may face patients. The process was recursive, and the last stage of the six-step process to finalise and name the themes was carried out through review and iteration with the project team. Any disagreement was discussed and resolved within the study team as part of the analysis process.

Quantitative analysis was performed for specific questions that were asked to all patients interviewed, to assess whether they had been aware of the missed appointment and if so, what method of communication they had received. This quantitative analysis involved calculating the percentage of patients interviewed who had been aware of the appointment, and the percentage of patients who received each communication type.

### Behavioural science frameworks

As discussed above, the TDF was chosen as a behavioural science framework to provide a theoretical basis to the reasons contributing to missed appointments. It aims to provide a lens to explain the different influences on human behaviour, such as cognitive, social and environmental [29]. The themes were mapped onto the relevant 14 TDF domains in a deductive approach, to identify behaviour change components.

### Researcher characteristics and reflexivity

The research team was comprised of a multidisciplinary team of designers, behavioural science researchers, policy fellows and patient and public involvement specialists. This diversity of thought and experience led to strong collaboration and communication from various team members throughout the study. The involvement of a public involvement specialist ensured the championing of the patient, public and carer perspective throughout the study, such as recruiting a public steering group (PSG) who were from minority ethnic groups or living in deprived areas to influence the study. Members of the PSG provided feedback on the discussion guides and reviewed project outputs. The researchers who conducted and analysed the interviews were aware of the potential of introducing bias through their own experiences and perspectives. To mitigate this, the discussion guides were used during the interviews and team input and reflections were sought throughout the thematic analysis and framework development process.

## Results

### Patient demographics

A total of 26 patients who had missed their first outpatient appointment at one of the five clinical specialties, or a family member, were interviewed. Twenty-two (84.6%) patients interviewed had their ethnicity recorded in the EHR as “Black”, “mixed” or “other”. Nine (34.6%) patients lived in an area with the highest level of deprivation (IMD quintile 1). For six patients, the interview was conducted with a family member who managed their healthcare, such as a child or grandchild. In three of these cases, the family member also translated directly to the patient to allow them to participate. Further detail on these patient demographics is shown in Table 1.

Initially, interviews were planned to last 10 to 15 min with patients. However, given the nature of these unscheduled calls, the team found that patients were often not available or unprepared to talk in-depth about appointment barriers, as they may be at work or carrying out other responsibilities and unable to take the call. To overcome this challenge, the data collection approach was modified to include an initial screening call to inform patients about the interview and decide a time when they would be called back for a longer interview. This allowed the patients to prepare for the interview and schedule it for a time that was suitable for them. One of these scheduled interviews lasted 30 min for which the patient was reimbursed for their time as per best practice guidelines [30].

**Table 1:** Overview of patients interviewed, describing their ethnicity and deprivation level of where they live

Characteristic	Ophthalmology	Gastroenterology	Colorectal surgery	Plastic surgery	Cardiology	Total (%)
**Ethnicity**						
• Black	2	1	-	3	3	9 (34.6%)
• Other	2	1	4	1	1	9 (34.6%)
• Mixed	-	2	-	-	2	4 (15.4%)
• Asian	1	-	1	-	-	2 (7.7%)
• White	-	1	-	-	1	2 (7.7%)
**IMD**						
• 1 (Most deprived)	2	3	3	-	3	9 (34.6%)
• 2	3	-	1	3	2	9 (34.6%)
• 3	-	1	-	1	2	4 (15.2%)
• 4	-	1	-	-	-	1 (3.8%)
• 5 (Least deprived)	-	-	1	-	-	1 (3.8%)

### Staff demographics

Eleven staff members from four of the five clinical specialities and the Patient Service Centre were interviewed, as shown in Table 2. It was not possible to interview staff from the ophthalmology service due to staff unavailability during the study. Of these 11 staff participants, two members from cardiology and two members from the PSC were shadowed to gain a more detailed understanding of the day-to-day appointment booking process. Interviews lasted 30 min to one hour.

**Table 2:** Overview of staff interviewed and observed from the five clinical specialties and Patient Service Centre

Staff area	Number of staff interviewed	Number of staff observed
**Clinical specialty**		
Cardiology	4	2
Gastroenterology	2	-
Colorectal surgery	1	-
Plastic surgery	1	-
Ophthalmology	-	-
**Outpatient support services**		
Patient Service Centre	3	2

### Thematic analysis

The thematic analysis and triangulation of all qualitative data led to the development of seven high-level themes, each with specific subthemes (Table 3). These themes represent the barriers and systemic issues that patients at risk of inequity, based on their ethnicity or deprivation, may face that can result in missed first outpatient appointments. After the initial coding, a second researcher reviewed 10% of patient interview notes. Most of the codes generated and compared by each team member were consistent, with any discrepancies discussed and added or removed. There were disagreements in the research group around naming of the subthemes and these were resolved through group discussion. For example, insights and quotes related to the subtheme ‘conflicting information’ were initially included within the ‘unclear or insufficient information’ subtheme, but through discussion it was determined that this was a separate subtheme. The TDF domains were mapped onto each subtheme, which highlighted the aspects of behaviour change that could support individuals to attend their appointments. The broad range of barriers and systemic issues identified in Table 3 reinforced the existing literature by demonstrating the variety of factors that influence the ability of patients to attend.

**Table 3:** Barriers and issues related to missed outpatient appointments mapped onto theoretical domains framework

Theme	Subtheme	Theoretical domains framework
Communication Factors	Late communication	Knowledge
	Lack of communication	Knowledge
	Unclear or insufficient information	Knowledge
	Communication variability	Knowledge
	Conflicting information	Knowledge
	Digital literacy	Skills
	Language	Environmental context and resources
	Family support	Social influences
Communication Methods	Phone communication	Knowledge Memory, attention and decision processes Environmental context and resources
	Letter communication	Knowledge Memory, attention and decision processes Environmental context and resources
	Email communication	Knowledge Environmental context and resources
	Physical reminder	Memory, attention and decision processes
Healthcare System	Delays	Environmental context and resources
	Service capacity	Environmental context and resources
	Private healthcare	Beliefs about consequences Environmental context and resources
	Perception of NHS	Environmental context and resources Emotion
	GP point of contact	Environmental context and resources
System Error	Hospital cancellation	Environmental context and resources
	Staff missed appointment	Environmental context and resources
	Patient re-scheduled	Environmental context and resources
	Other error	Environmental context and resources
Transport	Patient transport service	Skills Environmental context and resources
	Public transport	Skills Environmental context and resources
	Access needs	Skills Environmental context and resources
	Other transport options or issues	Skills Environmental context and resources
Appointment Factors	Appointment time	Environmental context and resources Social influences
	Appointment type	Beliefs about consequences
	Ability to self-book	Memory, attention and decision processes
	Ability to reschedule	Knowledge Environmental context & resources Motivation and goals
Personal Factors	Caring responsibilities	Environmental context and resources Social influences
	Memory and decision-making	Memory, attention and decision processes
	Beliefs about consequences	Beliefs about consequences
	Multiple appointments	Knowledge Environmental context and resources
	Illness	Environmental context and resources
	Time off work	Social influences
	Other personal issues	Environmental context and resources Social influences

#### Theme 1: communication factors

Patients identified barriers they faced which limited the effectiveness of communication about their outpatient appointments. Even if patients received the communication on time, the lack of or unclear information provided within the communication modality meant that patients did not have the information needed to attend their appointments. The focus on support from family members to help with appointment communication was also evident.

*“They told me that it was the second time they tried to arrange the appointment for me, but I didn’t receive anything for the first appointment. I don’t know – I didn’t receive anything​.” Patient 11; Ethnicity – Other; IMD – 2*.

*“Sometimes people use their number for more than one occasion. They don’t give you the details in the text message. I use the phone for my mum and dad – I don’t know who the appointment is for.” Patient 14; Ethnicity – Other; IMD – 2*.

*“My dad gets the letters, but sometimes he can be forgetful. I get all the texts and phone calls” – Daughter of Patient 5; Ethnicity – Black; IMD – 1*.

Staff interviews corroborated patient insights, by highlighting the provision of unclear or insufficient information and adding that there is an inability within the system infrastructure to provide tailored and accessible communication about appointments. Staff described how these issues result in patients struggling to access or understand appointment communications. Language was highlighted as an issue related to written and phone communications, such as the inability to send communication in different languages and the lack of provision of translators for phone communications with patients.

*The automated letter that is sent from [name of hospital] doesn’t have the specific information needed for certain scans, such as instructions on fasting – Staff members 1 and 2*.

*So much information provided in the letters and patients cannot pull out the key information – Staff members 1 and 2*.

*The appointment information provided is not in any other language than English. Even if on the patient record their spoken language is not English, the documents cannot be sent in that language – Staff members 1 and 2*.

#### Theme 2: communication method

Patients identified issues related to specific communication methods used by healthcare services to provide information about their outpatient appointment, specifically phone, email, and letter reminders. Phone communication was a challenge as patients may not have access to a charged and working phone, although calls or texts close to the time of the appointment were seen as helpful as reminders. While some patients preferred letters as they did not require digital literacy skills or equipment, others found that they were easy to misplace or had issues with delivery. Emails were preferred by individuals with strong digital literacy skills as a source of immediate information, but others had setting up email reminders and access challenges.

*“I like the letters, sometimes I lose my telephone, or it’s charging – sometimes there’s a problem with the telephone​” – Patient 12; Ethnicity – Asian; IMD – 1*.

*“The day before the appointment they ring me and say my appointment is the next day. I also get a text… To be honest with you, it’s got to be 2 or 3 days before the appointment so I can make space for that. I must admit I do forget” – Patient 24; Ethnicity – Black; IMD – 2*.

*“I have issues with the post to do with where I live. It’s not just the post that’s unreliable – because it’s a shared post box so they don’t always get to me” – Patient 21; Ethnicity – Black; IMD – 2*.

*“They’ve done an email for me – I haven’t used it myself, I’ve written it down somewhere. They have to explain to me how to use” – Patient 24; Ethnicity – Black; IMD – 2*.

Systemic issues identified by staff members related to how text reminders are sent to patients from an NHS phone number that they cannot call back, which is frustrating for patients as they cannot make enquiries or reschedule directly. Letters were most frequently mentioned by staff as a failed communication method, highlighting that patients often say that they have not received the appointment letter.

*Texts come from an NHS number that they can text back on but can’t ring. When they do ring it back they just get a general voice message that says they’ve gotten a call from [name of Trust] – Staff member 11*.

*There is the option to print an automated letter when the appointment is made and this request is sent to [name of hospital] where it’s printed. However, the service doesn’t trust this system as often the patients say that they don’t receive these letters. Therefore, the service staff usually print their own letter – Staff members 1 and 2*.

Postal strikes mean sometimes people don’t get a letter – staff member 5

#### Theme 3: healthcare system

The wider healthcare system, referring to the way in which healthcare services are set-up and organised, beyond the control of the outpatient services at the study site, was identified as a significant source of barriers by patients. Appointment delays and service capacity issues meant that several patients had chosen to seek private healthcare services, without being removed from the NHS waiting list. Several patients noted their negative perceptions of the outpatient experience and wider NHS services. Patients identified the lack of action or communication by their general practitioner (GP) to facilitate their outpatient appointment as an additional issue.

*“If you’ve not seen someone for 2 years you need to ask the person if they’ve been seen elsewhere. I’ve been seen privately – that’s another person off the waiting list” – Patient 4; Ethnicity – Black; IMD – 2*.

*“No reminder text – nothing, it was a shambles. [name of Trust] is a big Trust, they should be doing this – sending a reminder​” – Patient 19; Ethnicity – Black; IMD – 4*.

*“Referred by the GP, they didn’t give any guidance about what to expect – didn’t communicate anything with that regard” – Patient 4; Ethnicity – Black; IMD – 2*.

*“My GP doesn’t do anything for me. After one and a half years they sent me to MRI. Then they refer me to the hospital and nothing. The NHS haven’t done anything for me” – Patient 14; Ethnicity – Other; IMD – 2*.

#### Theme 4: system error

Despite some patients intending to attend their appointment, rescheduling, and even been present waiting for their video or phone appointment, errors within the administrative system led to some patients being incorrectly recorded as DNAs. In certain cases, this led to patients being discharged from the outpatient service without having the opportunity to attend their first appointment. Other patients said that the missed appointment had been cancelled by the hospital beforehand.

*“I was aware but I didn’t receive any call from them. I rang them and they said they’d give me a call on the day but I didn’t receive any call ​” – Patient 26 talking about a phone appointment; Ethnicity – Black; IMD – 3*.

*“They kept on pushing appointment forward, the appointment was on video. Eventually the date they gave me I joined the link, was waiting in the lobby for ages and no one came. My experience has not been great” – Patient 4; Ethnicity – Black; IMD – 2*.

*“Yeah, I was aware of the appointment, but I was also aware that the appointment was booked for the 31st March and so I didn’t attend. It was initially booked for the 9th and was changed to the 31st. I took the day off work just to attend the appointment on the 31st. On the 10th March I got a letter addressed to my GP and myself that I’ve been discharged from the service because I didn’t attend the appointment. But actually the appointment was rebooked for a different date on the 31st March. It’s bad that it was rebooked and I was discharged” – Patient 19; Ethnicity – Black; IMD – 4*.

These system errors were also highlighted in staff interviews, where staff shared that they could occur due to the complex administrative process behind appointment booking and communications.

*Sometimes the clinics make a mistake and discharge a patient when they have only DNA’d once – Staff member 3 talking about the Trust policy, which states that patients may miss an appointment twice*.

#### Theme 5: transport

Patients reported challenges when travelling to appointments, including issues with the patient transport service, public transport options, and general access needs. Difficulties related to the existing patient transport service included early pick-up times that interfered with their health needs or caring responsibilities, issues with getting collected by the service and delays in being transported home. While some patients preferred to use public transport, access needs meant that for others this was not possible.

*“Either you have to go hours before your appointment and then hang around or coming back is even worse. I go with my dad – I take him and bring him back. I go past the patient lounge and it’s ridiculous – it’s just not an option and I would like it to be” – Daughter of Patient 5 talking about the patient transport service; Ethnicity – Black; IMD – 1*.

*“I’m 60 now, but I cannot use the underground any more. When I go on the underground I can’t breathe – It doesn’t agree with me at all so I have to use the buses. I have to leave in time because of roadworks and that… can’t get the train or the tube. I’ve got a freedom pass” – Patient 24; Ethnicity – Black; IMD – 2*.

Staff found it challenging to support patients with the patient transport service, as it must be booked directly by the patient through an external organisation. The study site has specialist national services, so they noted that this is particularly an issue for patients travelling long distances. They also highlighted that some patients struggle to attend early morning appointments as free transport passes only work within certain times.

*Sometimes a transport request is in the referral letter but all we can do is give the transport number. The patient has to book it themselves – Staff member 3*.

*[Name of Trust] has some specialist cardiology services that are national, such as the national pulmonary hypertension service. This means that patients travel very long distances to attend appointments and may not be able to organise transport or are not eligible for the patient transport service – Staff members 1 and 2*.

Bus pass doesn’t start working until 9.30am – staff member 3

#### Theme 6: appointment factors

Many patients had preferences for their outpatient appointment time and type, due to health needs, work, or caring responsibilities. However, this often is not reflected in the appointment given and as a result they cannot attend their scheduled appointment. Patients also reported that the appointment booking processes were inflexible or inaccessible, thus it was difficult for some patients to book or reschedule an appointment that suited their needs. Different appointment formats, such as video calls, intended to offer more flexibility were not deemed appropriate or useful by some patients.

*“Generally I just receive an appointment, which is not always ideal for me – I don’t get to choose the appointment time​… Sometimes they send me an appointment for 10 in the morning and I can’t go because I’m with my mum” – Daughter of Patient 5; Ethnicity – Black; IMD – 1*.

*“The problem right now, the letters that come there’s no direct number to call. Most of the time when I ring it goes to a central place. They can ring me but I can’t ring them, can’t get to speak to any of them” – Patient 24; Ethnicity – Black; IMD – 2*.

*“If it’s video it’s not helpful – I don’t want to take time off work to attend a video consultation” – Patient 4; Ethnicity – Black; IMD – 2*.

Staff shared that they thought appointments provided over the phone and during the weekend supported patients to attend. However, they acknowledged that there are difficulties for patients attending weekend appointments when other outpatient support services are not available.

*There are lots of weekend clinics now which should give more time options for people to choose from – may be easier to attend – Staff members 1 and 2*.

*More weekend clinics ongoing, but the call centre isn’t open at the weekend. So, if someone is sick over the weekend and can’t make their appointment, they can’t cancel and will show up as a DNA – Staff member 11*.

#### Theme 7: personal factors

Many personal factors were reported by patients that led to challenges in attending their appointments. Although some patients wanted to attend, they were forced to miss their appointment as it was scheduled at times when they had existing responsibilities or commitments. Unexpected circumstances which prevented patients from attending appointments also arose and the inability to reschedule, as highlighted above, meant that the appointment was not utilised. Several patients were sick and unable to attend or reported issues with taking time off work to attend a hospital appointment. Some participants struggled to remember to attend their appointments without reminders or keep track of multiple appointments.

*“I was aware but I couldn’t make it – I had family issues​” – Patient 13; Ethnicity – Black; IMD – 1*.

*“I can’t go because I’m with my mum… I need someone to sit with my mum when I go” – Daughter of Patient 5; Ethnicity – Black; IMD – 1*.

*“Two people were sacked from the company and I couldn’t take a day off from work” – Patient 11; Ethnicity – Other; IMD − 2*.

Staff supplemented these insights, noting that certain specialties predominantly have older patient groups who may find it challenging to remember or attend hospital appointments, particularly if they are attending multiple services or sites.

*In cardiology a lot of the patients are older, this means that they may forget about their appointments or often have a lot of appointments to keep track of – Staff members 1 and 2*.

*If a patient has two appointments – one in morning and one in afternoon, they may just decide not to go to both. Alternately, people usually have multiple diagnostics appointments and sometimes they are not able to be booked into them all at the same time – Staff members 1 and 2*.

### Staff observations

Two members of the cardiology team and two members of the Patient Service Centre were shadowed as part of their interview. Data gathered during the observation were recorded alongside the interview notes to create a broader understanding of the systemic issues affecting outpatient attendance. Patients called the PSC for multiple reasons, such as to reschedule their appointment due to personal issues or to reschedule an appointment that they had missed without being aware, linking to the “personal factors” and “appointment factors” themes. By observing PSC staff take inbound calls, insights were gained about the appointment booking and rescheduling process, such as how many manual and complex processes are involved. These processes involve many individuals and departments and can be challenging for the PSC to coordinate on behalf of the patient given that they are highly manual and rely on human input, which related to the “healthcare system” and “system errors” themes.

### Quantitative analysis

Eighteen of the 26 patients (69.2%) interviewed said they had been aware of the appointment they missed. When asked about how they had been made aware of the appointment, nine received a letter, two received a text, one received an email, two received both a letter and a text and one received an email and a text. Three patients did not answer the question, or their answers was unclear. Eight (30.8%) patients responded that they were not aware of the missed appointment.

## Discussion

### Key findings

This study highlighted the key barriers contributing to DNAs in the patient groups most likely to be affected by inequity of access due to their ethnicity or deprivation. These barriers included communication factors and methods, the healthcare system, system errors, transport, appointment, and personal factors. While these barriers were identified primarily through patient interviews, they were triangulated with and supported by qualitative data obtained through discussions and observations of healthcare staff. The quantitative analysis of patient awareness about their missed appointment helped showcase certain barriers, such as issues with receiving appointment communications.

The study highlights the importance of identifying barriers for patient groups experiencing inequity as they may experience unique challenges that must be addressed to support appointment attendance, such as negative cultural perceptions of NHS services or language needs. The onus of this is on healthcare services to adapt their practices, tailor communication methods and provide support to specific communities. While some barriers faced by individuals can be influenced by practical offers such as reimbursement of travel costs or interpretation services, it is imperative that tailored, accessible hospital communications to patients about upcoming appointments are implemented.

### Evidence in context of existing research

This work builds on the literature focusing on patient groups experiencing inequity, to identify the barriers preventing patients from attending scheduled outpatient appointments [20]. Our study findings are consistent with previous literature suggesting that the reasons behind missed appointments are complex and often intersect with and compound each other and that there are specific issues affecting populations experiencing inequity [20]. Patient forgetfulness is commonly cited as a primary reason for non-attendance [31, 32] along with transport issues [33, 34]. Compared to other studies [35], our findings had less emphasis on barriers related to associated cost of attending and loss of earning potential, which may have been due to the nature of the questions in our interview guide. However, our work builds upon this to further elaborate on these specific barriers and identify systemic issues, for example healthcare system errors, that can result in the incorrect recording of a missed appointment. Given its use in implementation research [26], the TDF adds evidence to the existing knowledge base by aiming to provide practical, implementation support when appropriate solutions are designed for barriers in the future. This study involved interviewing patients and staff from outpatient services in a London Trust, therefore providing evidence for a specific region and for communities who are often underrepresented in research.

### Study strengths

There are multiple strengths that supported the conduct of this study and the validity of its findings. The dual study aims focusing on patient and staff perspectives ensured that a holistic and accurate representation of the patient barriers and systemic factors to missed appointments were identified. The process of completing interviews with staff and patients in parallel along with staff shadowing allowed barriers raised by patients to be understood in greater detail by investigating them from the system perspective with healthcare staff. Asking patients questions about appointment awareness at the same time as the interview allowed for quantitative data to be gathered in parallel. The research team’s responsiveness to adapt the patient interview process ensured that interviews were carried out at a time suitable to the patient and gave them adequate time to prepare.

Using the TDF to develop the discussion guide and map onto the framework of barriers and issues allowed the research team to understand potential supports required in the future to address these factors. While some TDF domains, such as memory or knowledge, can be addressed through co-designing practical interventions that address these barriers, many of the issues related to the wider environmental context and resources available to patients. This provides evidence that certain barriers are outside the control of individual patients, and, therefore, future healthcare and wider societal systems are required to change to support patients, particular from underserved groups, to attend appointments.

### Study limitations

The present work has several limitations. Despite our study population including patients from minority ethnic groups, no translation services were offered to patients during the phone interviews. This meant that individuals unable to be interviewed through English were excluded from the study. As a result, fewer individuals currently facing language barriers when interacting with healthcare services were interviewed and their experiences not actively included.

A similar structural limitation of the study was carrying out the interviews during conventional working hours. This meant that many of those at work would not have answered the phone when called, and their perspectives were missed. As a result, our findings may have lacked perspectives from those who work at jobs or industries with traditional hours, and instead captured perspectives of those who are retired, unemployed, or work flexible hours.

An inherent selection bias in our work is the issue with poor quality contact information for patients in NHS systems. While some patients we attempted to contact were found to have inactivated phone numbers, others may have not answered the phone due to changing phone numbers and the EHR not being updated. Two studies on text message reminders for cervical screening showed that 36.4% and 38% of patients had an up-to-date mobile phone number recorded in their clinical record, with this varying by age and IMD subgroups of participants [36]. As a result, we were unable to interview patients who had incorrect contact information recorded, and therefore who were also more likely to have not received information about their missed outpatient appointment. In addition, data on other patient demographics such as age and education level were not collected, which may have provided information on potential confounding factors for some of the barriers identified.

Due to the study being carried out in the chosen five clinical specialties, we anticipate that findings are transferrable to similar outpatient clinical specialties experiencing the same pressures and with similar patient cohorts. However, the study setting is a large NHS Trust in London’s city centre, meaning that the findings may not be transferable to other settings, such as those in rural environments or healthcare providers outside the NHS.

### Opportunities for future work

Several opportunities exist to replicate this work in other settings and develop future solutions to the barriers identified. By repeating this work in other specialties or study settings, this would allow more specific insights to be developed for populations or environments. To gain additional insights, further studies could include interpretation services or carry out interviews in local community spaces. In addition, further work should be performed to co-design interventions with public members to address the barriers identified in this study. This would ensure that acceptable and feasible interventions are developed alongside healthcare services to support individuals facing inequity attend their appointments. In this future work, public members should be involved from the beginning to address potential limitations. This work, and future initiatives, are supported by the NHS goals of reducing health inequalities [37], particularly in healthcare provision [38].

## Conclusion

Patient groups experiencing inequity of outpatient access based on their ethnicity or the deprivation of where they live were found through patient interviews to face a variety of barriers to attending their appointments, such as communication needs and preferences, transport, appointment booking processes and systemic issues such as negative perceptions of wider NHS and health services. This was supported by staff interviews and observation to expand on the causes of missed appointments from a service perspective. Several patients were not aware of the appointment they had missed, highlighting barriers related to appointment awareness. Health services need to provide additional tailored support to individuals to attend their outpatient appointments, as opposed to a “one size fits all” approach. The application of a behavioural science framework provides a strong basis for co-designing future interventions to address these barriers.

### Electronic supplementary material

Below is the link to the electronic supplementary material.

Supplementary Material 1

Supplementary Material 2

## Data Availability

The discussion guides used in this study are provided as supplementary information files. The data generated or analysed during this study are available from the corresponding author on reasonable request.

## Footnotes

1 Ethnicity data collected by NHS organisations uses national definitions where a limited number of ethnicity categories are provided as options. For example, the high-level category “other” includes two ethnicity categories – “other ethnic groups – Chinese” and “other ethnic groups – any other ethnic group” [22].

## Abbreviations

COVID-19: Coronavirus Disease 2019
DNA: did not attend
GP: general practitioner
HER: electronic healthcare record
HRA: Health Research Authority
IMD: index of multiple deprivation
NHS: National Health Service
PSC: Patient Service Centre
PSG: public steering group
TDF: theoretical domain framework
UK: United Kingdom
