# Supplementary material for: Identifying barriers to outpatient appointment attendance in patient groups at risk of inequity: a mixed methods study in a London NHS trust
Source: BMC Health Serv Res. 2024 Apr 30;24:554. doi: 10.1186/s12913-024-10947-8 (PMC11061980; doi:10.1186/s12913-024-10947-8)
Supplement: Supplementary file 1 — Supplementary Material 1 [file 12913_2024_10947_MOESM1_ESM.docx]

**Discussion guide - Patients**

**Format**: We will call (20-30) patients across 5 outpatient clinics who have not attended their appointment

**Aim**: Understand the appointment booking pathway and the issues and barriers to patient attendance

**Section 1: Welcome**

Call patient’s number:

***“Hello, my name is Alice, and I am calling from [Name of Trust] to understand how we can improve our services. Am I speaking to...?”***

If the person answering is not the patient it is important to maintain confidentiality and data protection, and not to share this personal patient information with anyone else.

***“I am calling regarding a hospital appointment for [insert patient name] and due to patient confidentiality, I need to speak to them directly. Could I speak to them now or would I be able to speak to you about this with the patient’s consent please?”***

If consent is given by the patient to speak to the respondent, continue to Section 2: Introduction & Overview.

If consent is not given, thank respondent and end call, or if patient is not available currently, note down when the patient would be free to talk and call back at this time.

**Section 2: Introduction & Overview**

Confirm patient details

***“I just need to confirm that I’m speaking with the right person, can you confirm your/the patient’s (if speaking to someone of their behalf) full name, date of birth and postcode please?”***

Ensure this corresponds to the details on information that we have been provided by the admin team.

Provide introduction of researcher and brief outline of project aims.

***“We are contacting patients with recent hospital appointments to see if there are any ways we can improve access to our service. We are interested to speak with patients to understand what difficulties they might experience making it to their hospital appointments. This will take around 5 minutes.***

***I will be asking you questions, and my colleague Fiona will be taking notes while we’re talking.***

***These notes will all be anonymised and nothing you say will be linked back to your name and affect your care at the hospital.***

***Are you happy to continue and answer a few questions?”***

Confirm participant is happy to proceed.

If they cannot talk at that time, but would still like to be interviewed, make note of when they would be free to be called back.

***“Please feel free to ask me to repeat any questions or ask any questions yourself at any stage”***

**Section 3: Interview**

Giving information:

“***On our system it shows that you had an appointment scheduled on:***

- ***Provide date of appointment***
- ***Provide time of appointment***
- ***Provide appointment specialty***

*Section 1: reason for non-attendance*

1. Were you aware this appointment was made?
2. How did you know that the appointment was made? E.g. what communication methods did they received from the Trust
3. What made it difficult for you to come?
   *Prompt: Probing questions to establish route cause*
4. Have you been to an appointment at hospital before? What was different this time that meant you couldn’t come to the appointment?

*Section 2: Making the appointment*

1. Tell us about the process for making the appointment or learning that you had an appointment.

- What did that involve?
- How did you become aware of the [insert clinic name] clinic?
- What did the process for getting referred involve?
- Who did you see or speak to?
- What systems e.g. websites or forms did you have to use?
- What was good about this experience? And bad?

*Section 3: Waiting for the appointment*

Tell us about the time leading up to the appointment

- How long were you waiting for the appointment?
- What communications did you receive? Were you reminded? If so, how, and when?
- How did you find this entire process?
- Did anything change for you whilst you were waiting that affected you attending the appointment?

*Section 4: Desirable future state*

1. Was there anything that could have been done better to help you attend this appointment?
2. In an ideal world, how would the referral (appointment) have been managed differently?
   *Prompt: How could the process of making and attending appointments at the [insert] clinic be improved?*

*Section 5: Rescheduling the appointment*

1. Have you re-scheduled the appointment? Tell us about how you did that
   *Prompt: Was there anything that made this difficult for you?*

- [If they haven’t rescheduled], what do you think will happen next?

If they ask to reschedule:

- We are unable to reschedule your appointment but we encourage you to give them the outpatient appointment booking team a call, the number is at 020 3313 5000.

If a distressing topic is discussed:

- Encourage participant to contact Patient advice and liaison service (PALS) and complaints team [Hospital PALS number: 020 3312 7777, Monday to Friday, 10.00-16.00. More [contact info](https://www.imperial.nhs.uk/patients-and-visitors/help-and-support/feedback-compliments-and-complaints/pals) for PALS at other Hospital sites]

*Section 6: Close*

1. Is there anything we haven’t talked about that you would like to share?

   ***“Thank you so much for your time today, that is all the questions we would like to ask, would you like to ask us anything?***

***We hope to use the information that we have learned through these conversations with patients to improve the service.***

***Reminder: no personal identifiers or any personal data will be used, just anonymised quotes. Your name will not be linked to anything you say.”***

Frequently asked question (FAQs)

1. ***Why have you called me about these questions?***

We are collecting information from patients that our records show have recently missed their appointments and we are particularly focusing on patients from ethnic minority backgrounds and live in more deprived areas as previous data analysis consistently shows that patients from these backgrounds are more likely to miss their appointments. We are trying to improve our appointment service specifically for these patients through a better understanding of their needs, and therefore gathering information from patients like yourself would be very helpful to do this.

1. ***What personal information is being recorded about me?***

The personal information recorded about you will be your ethnicity and the level of deprivation in the area you live in, however nothing that will allow you to be personally identifiable i.e., name or contact details.

1. ***Where are my responses being recorded?***

These will be anonymised and recorded electronically by the team.

1. ***Will my answers be on my medical record/will my appointments or treatment in the future be affected by my answers?***

None of the information you give will be put on your formal medical record. Your appointments or treatment will not be affected by the responses.

1. ***Who else will have access to this information?***

It will be a team at the Trust and Imperial College London that will analyse and synthesise the data. People who have access to this are trained to protect patient data, maintain confidentiality, and will only use this information for the purposes of improving our service.

1. ***How will my answers be used?***

They will be collated with other patient responses to identify change ideas which may

improve our outpatient appointment service so more patients can attend their appointments. What we do next depends on the responses we collect from patients and how feasible the changes are for each service to make e.g., more reminder messaging, creating more clinic times out of hours.

1. ***I would like to re-book my appointment at X clinic. Can you do this?***

We cannot re-book your appointment ourselves since we are not storing any of your personal identifiable information. However, we can share the contact information for the outpatient appointment booking team at 020 3313 5000 to allow you to re-book it.
