# Supplementary material for: Identifying barriers to outpatient appointment attendance in patient groups at risk of inequity: a mixed methods study in a London NHS trust
Source: BMC Health Serv Res. 2024 Apr 30;24:554. doi: 10.1186/s12913-024-10947-8 (PMC11061980; doi:10.1186/s12913-024-10947-8)
Supplement: Supplementary file 2 — Supplementary Material 2 [file 12913_2024_10947_MOESM2_ESM.docx]

**Discussion guide (staff)**

**Format**: Face to face interviews with staff at the Patient Service Centre (outpatient appointment booking service centre) and 5 outpatient clinics

**Aim**: Understand the appointment booking pathway and the issues and barriers to patient attendance

**Introduction & Overview**

- Introduction of researcher and brief outline of project aims
  *I am a design researcher at Imperial College, we are working with the trust to help them to understand the accessibility of their outpatient service*
- Plan for Session
  - Expected duration 10 minutes but it is ok to continue for longer if you have more to say
  - Explanation that one researcher will ask questions and the other will take notes, notes will be anonymised
- Confirm participant is happy to proceed
- **“Please feel free to ask questions at any stage”**

**“We will now move onto the interview, which will explore your experience of delivering outpatient services”**

**Interview**

*Section 1: Context*

1. What is your job role?
   *Prompt: What does that involve?*

*Section 2: Before making an appointment*

1. How do you become aware that an appointment needs to be made?

- Who do you communicate with?
- What tools and systems do you use?

*Section 3: Making an appointment*

1. Tell us about the process of making an appointment for a patient?

- Describe each stage
- What information do you need to have in order to make the appointment
- What tools or systems do you use?
- Who do you have to communicate with? How do you do that?
- What can make this process challenging?

1. What training have you received in relation to making appointments?
2. What, if anything, can go wrong during the process from referral to appointment?

- Are there any workarounds that you use?

*Section 4: Reasons for not attending*

1. Do you have any ideas about why patients may sometimes fail to attend their appointment?

*Section 5: Re-scheduling the appointment*

1. If a patient DNA’s (does not attend) what do you do?

- If you reschedule the appointment, what does that involve?

1. If a patient wanted to reschedule an appointment how would they do it? E.g., call or email
2. How is this process handled for a patient to re-schedule?

- Is the appointment line / email address manned all day?
- If not, can they leave a message?
- How quickly is the patient contacted back to re-schedule?

*Section 6: Ideal future state*

1. In an ideal world, if time and money were no object, how would the outpatient service change?
   *Prompt: How would the tasks you do change? How would the tools or systems be different?*
2. Has your team previously tried anything to help reduce patients not attending appointments?
   *Prompt: What was good about that? And bad?*

*Section 7: Close*

1. Is there anything we haven’t talked about that you would like to share?

   **“Thank you for your time today, that is all the questions we would like to ask, would you like to ask us anything?”**

**Reminder: no personal identifiers or any personal data will be used, just anonymised quotes.**
